# A novel protein chip for simultaneous detection of antibodies against four epidemic swine viruses in China

**DOI:** 10.1186/s12917-020-02375-7

**Published:** 2020-05-26

**Authors:** Yue Wu, Xudan Wu, Jing Chen, Jingfei Hu, Xiaobo Huang, Bin Zhou

**Affiliations:** 1 MOE Joint International Research Laboratory of Animal Health and Food Safety, College of Veterinary Medicine, Nanjing Agricultural University, Nanjing, 210095 China; 2 College of Veterinary Medicine, Sichuan Agricultural University, Chengdu, 611130 China

**Keywords:** Protein biochip, Simultaneous detection, Swine diseases, antibodies

## Abstract

**Background:**

At present, pig industry in China is faced with the complex situation of mixed infection caused by multiple pathogens. It is urgent to develop some new high-throughput molecular diagnosis assays to simultaneously detect pathogens or antibodies. Biochip array technology has made it possible to screen thousands of samples simultaneously; it has been twice named as one of the top 10 scientific and technological breakthroughs. Studies have reported encouraging results using protein biochips for detecting antibodies against avian infectious bronchitis virus and ruminant bluetongue virus, but the research of this technology for the diagnosis of swine diseases is still sparse.

**Results:**

In this study, a novel protein chip was developed that can simultaneously detect the antibodies of four important swine viruses as follow, classical swine fever virus (CSFV), porcine parvovirus (PPV), Japanese encephalitis virus (JEV), and porcine reproductive and respiratory syndrome virus (PRRSV). Four prokaryotic expression plasmids pET-32a-E2 of CSFV, −VP2 of PPV, −EDIII of JEV, and -N of PRRSV were induced by IPTG (Isopropyl β-D-1-Thiogalactopyranoside) and overexpressed in *E.coli*, respectively. The purified proteins were identified by Western blotting and then printed on epoxy-coated glass slides. The optimized parameters of this diagnostic chip showed that the spotting concentrations of E2、VP2、EDIII、N proteins were 0.2, 0.4, 0.4, and 0.4 mg/mL. The optimal primary and secondary antibody dilutions were 1:50 and 1: 600. Compared with the commercial ELISA (Enzyme-linked immunosorbent assay) kits, the positive and negative coincidence rates of this chip were 95.8% ~ 100 and 86.2% ~ 100%, as well as, no cross-reaction.

**Conclusion:**

This protein chip provided a fast, specific, and sensitive method for simultaneous detection of antibodies in clinical serum samples. Compared with traditional methods, this protein chip can monitor very small amount of serum.

## Background

With the increase in scale of pig production, diseases of pigs have come to have an enormous impact on pork producers and often on the economy of pork producing countries. China, the world’s largest pork producer, stands to shoulder very large economic losses from swine diseases [1]. In large scale production practices, epidemic disease problems can be divided into three general categories. First, the occurrence of mixed infections or secondary infections, both cause high rates of morbidity and mortality [2, 3]. Second, the overlap of disease syndromes, such as reproductive disorders, difficult breathing, diarrhea, fever, makes it difficult to identify a specific disease [4]. Third, under pressure from vaccination and antibiotic treatment, new strains of familiar pathogens, as well as new pathogens, are leading to large scale outbreaks [5–7]. These conditions make it necessary to develop diagnostic assays that can quickly and reliably screen large numbers of clinical samples. This will allow a savings of clinical manpower and material resources, as well as reduce the mortality and morbidity of pigs [8–10].

Biochip array technology has made it possible to screen thousands of samples simultaneously; it has been twice named as one of the top 10 scientific and technological breakthroughs [11]. The protein biochip is a novel application of the sandwich-type antibody-capture assays, such as ELISA, the fundamental difference is that the capture proteins are covalently attached to the surface of the biochip in an ordered array. The microarray format also enables a highly integrated analysis [12–14]. The development of multi-level biochips will enable high-throughput quantitative and qualitative diagnosis of important pig diseases and will help solve current diagnostic needs for large number of animal diseases [15]. Studies have reported encouraging results using protein biochips for detecting antibodies against avian infectious bronchitis virus and ruminant bluetongue virus, but the research of this technology for the diagnosis of swine diseases is still sparse [14]. To date, there are few commercial protein biochips available for use in pig diagnostics [16, 17]. Therefore, protein biochips should be developed to meet domestic demand and promote the healthy development of the pig breading industry in China.

In this study, four proteins, the E2 protein of Classical Swine Fever Virus (CSFV)VP2 of Porcine Parvovirus (PPV), domain III of the E protein of Japanese Encephalitis Virus (JEV), and the N protein of Porcine Reproductive and Respiratory Syndrome Virus (PRRSV), were used as capture antigens to develop a fluorescent based detection assay for simultaneous screening serum samples. After protein, buffer, and antibody parameters were optimized, we found that the detection rate for positive samples was above 95%, with no cross-reaction. This protein biochip has the advantages of quick and simple operation with high sensitivity and specificity.

## Results

### Expression and purification of fusion proteins

**Fig. 1:**
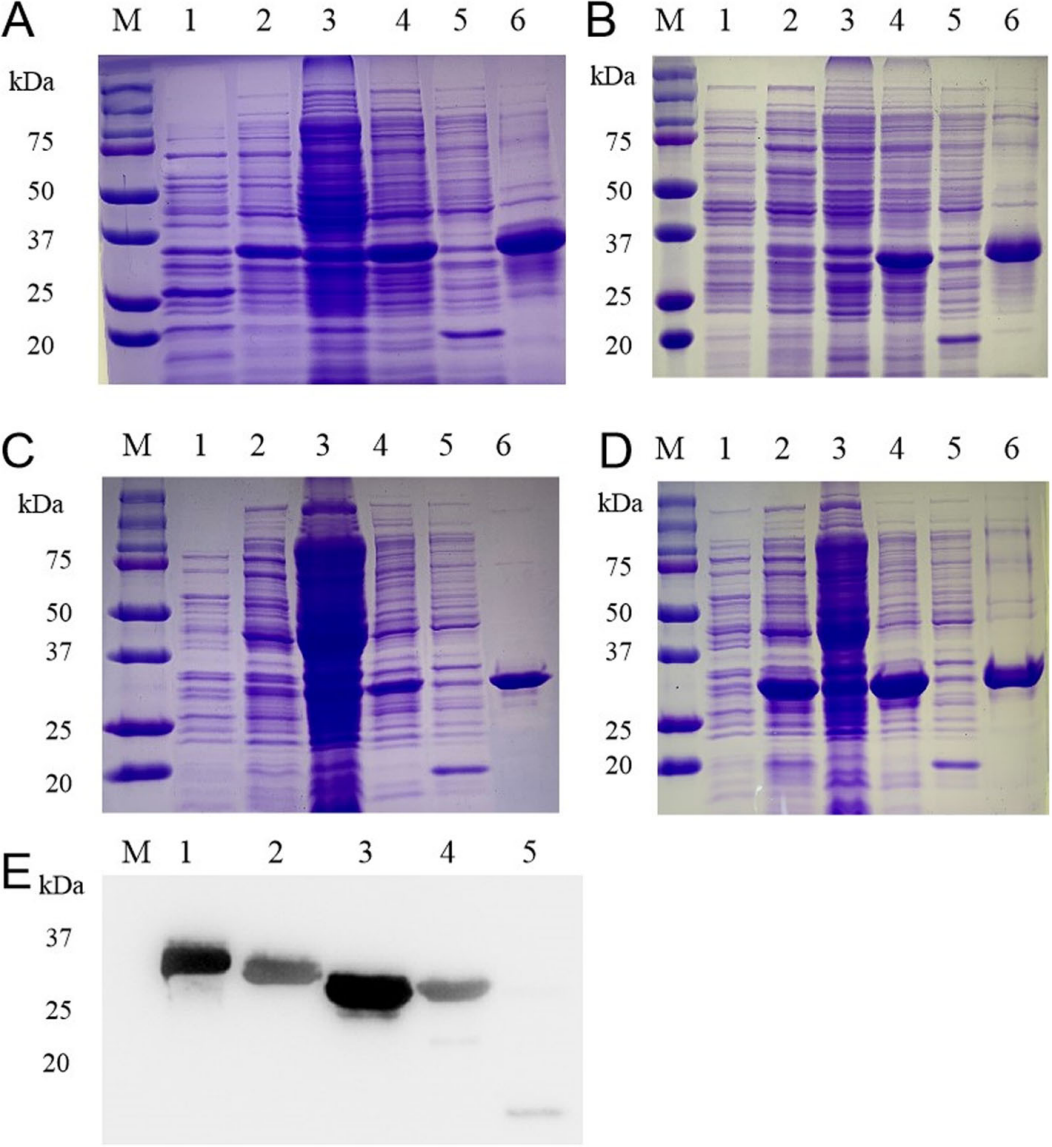
Expression and identification of fusion proteins. **a** SDS–PAGE analysis of CSFV-E2 protein expression and purification. 1- supernatant from uninduced bacteria; 2- supernatant from induced bacteria; 3- inclusion bodies from uninduced bacteria; 4- inclusion bodies from induced bacteria; 5- empty vector control; 6- purified E2 protein. **b** SDS–PAGE analysis of PPV-VP2 protein expression and purification. 1- supernatant from uninduced bacteria; 2-supernatant from induced bacteria; 3- inclusion bodies from uninduced bacteria; 4- inclusion bodies from induced bacteria; 5- empty vector control; 6- purified VP2 protein. **c** SDS–PAGE analysis of JEV-EDIII protein expression and purification. 1- supernatant from uninduced bacteria; 2- supernatant from induced bacteria; 3- inclusion bodies from uninduced bacteria; 4- inclusion bodies from induced bacteria; 5- empty vector control: 6- purified EDIII protein. **d** SDS-PAGE analysis of PRRSV-N protein expression and purification. 1- supernatant from uninduced bacteria; 2- supernatant from induced bacteria; 3- inclusion bodies from uninduced bacteria; 4- inclusion bodies from induced bacteria; 5. Control from empty vector; 6- purified N protein. **e** Western blot of bacterial lysates. After SDS-PAGE, the purified proteins were subject to western blotting using 6 × His-tagged mAb as secondary antibody.1. E2 protein; 2. VP2 protein; 3. EDIII protein; 4. N protein; 5. empty vector control

The four gene fragments were successfully amplified by PCR (Polymerase chain reaction), double-digested, and then cloned into pET-32a vectors. Plasmid constructs were verified by PCR and DNA sequencing and restriction enzyme digestion. SDS-PAGE analysis of the purification of transformed, IPTG induced bacteria, showed that the CSFV-E2 fusion protein (35 kDa) was expressed in the supernatant and inclusion bodies, while the PPV-VP2, JEV-EDIII, and PRRSV-N fusion proteins (30 kDa) were expressed only in inclusion bodies. These results demonstrate that each protein was expressed successfully and that high purity protein was obtained after Ni column purification (Fig. 1).

### Optimization of the protein chip assay

#### Antigen concentration

**Fig. 2:**
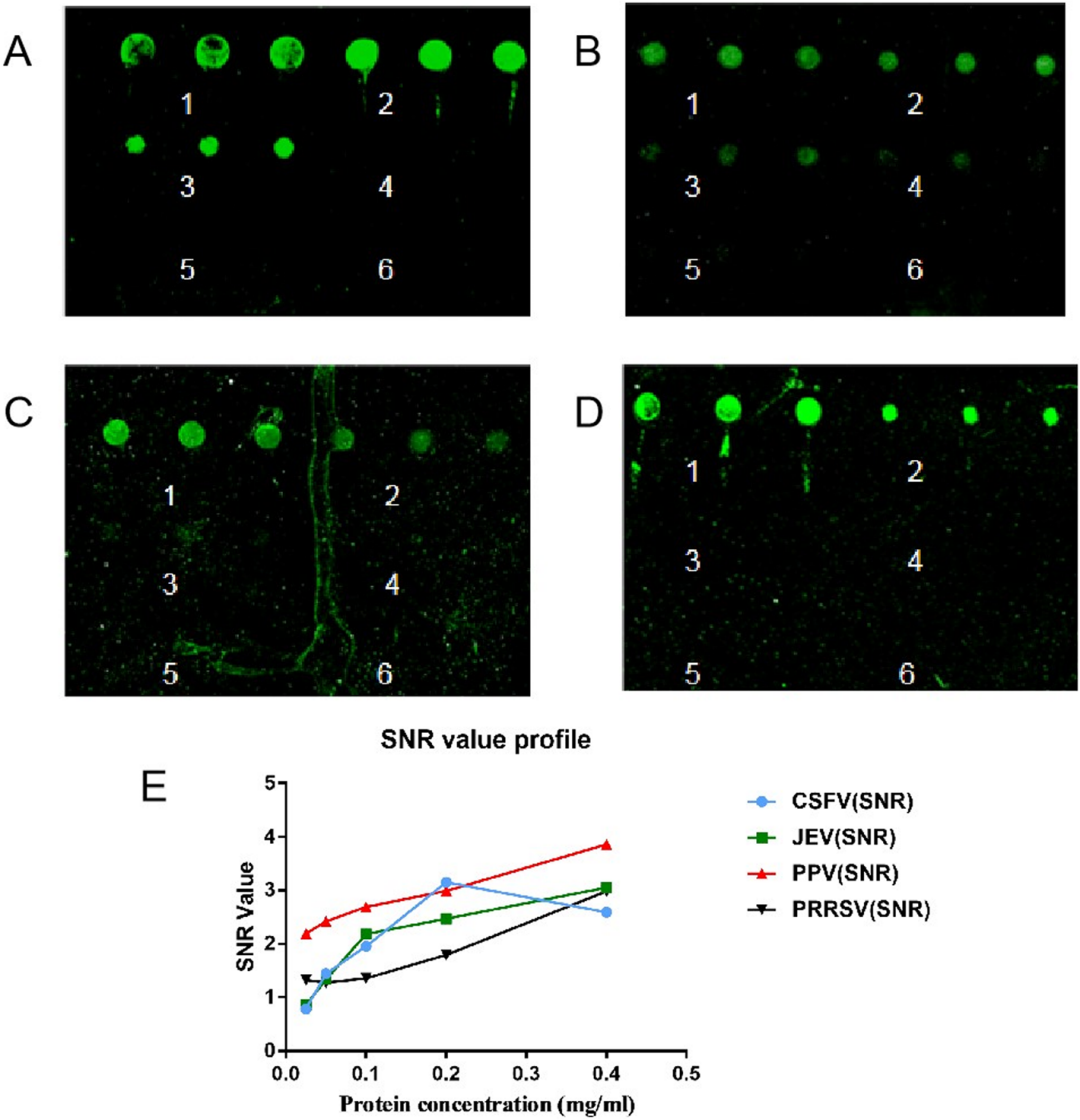
Optimal antigen concentration. Each fusion protein was spotted on the epoxy slides at concentrations of 0.4, 0.2, 0.1, 0.05, and 0.025 mg/mL in triplicate. Optimum concentration of **a** CSFV-E2, (B) PPV-VP2, **c** JE-EDIII and **d** PRRSV-N. Numbers indicate concentration 1, 0.4 mg/mL; 2, 0.2 mg/mL; 3, 0.1 mg/mL; 4, 0.05 mg/mL; 5, 0.025 mg/mL; and 6, negative control. **e** SNR profile for each protein

All proteins were diluted to 0.4, 0.2, 0.1, 0.05, 0.025 mg/mL. Scanning analysis showed that for the CSFV-E2 protein, 0.2 mg/mL was the optimal concentration, and for PPV-VP2, JEV-EDIII, and PRRSV-N 0.4 mg/mL was the optimal (Fig. 2).

#### Print buffer

**Fig. 3:**
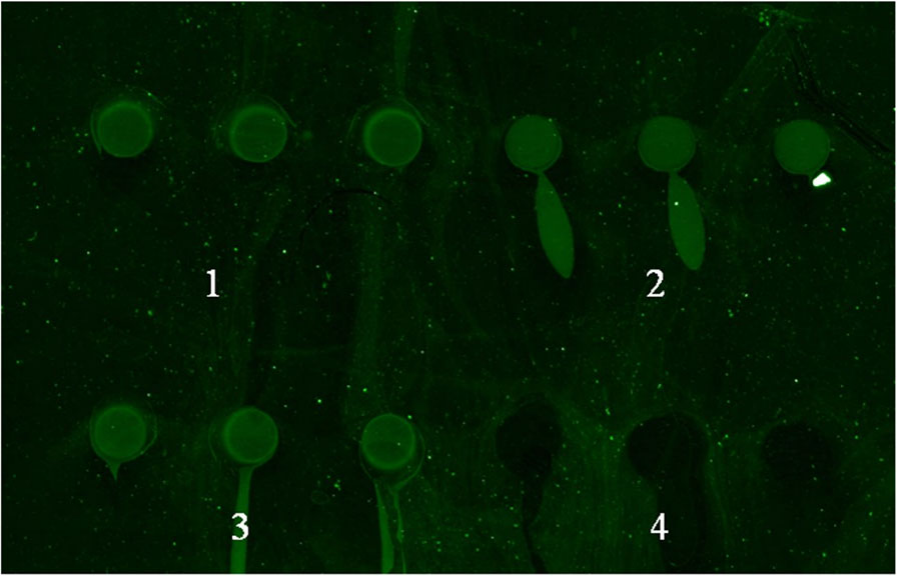
Optimal print buffer. The protein samples of CSFV-E2 were diluted with 40%vol or commercial print buffer, or 50%vol homemade print buffer to the optimal concentration and spotted on epoxy chip. 1, 50%vol commercial print buffer; 2, 50%vol homemade print buffer; 3, 40%vol commercial print buffer; 4, Negative control of BSA

The print buffers tested were 40%vol (volume) and 50%vol commercial buffer, and 50% homemade buffer, as well as the negative control of 2% BSA (Bovine serum albumin). 50%vol commercial buffer resulted in discrete fluorescence spots with little or no smearing. 40% commercial, and 50% homemade buffer resulted in spots with moderate to substantial smearing (Fig. 3).

#### Antibody dilution and incubation time

The optimal dilution for primary antibodies against CSFV-E2, PPV-VP2, JEV-EDIII and PRRSV-N was 50-fold with 1 h incubation time at 37 °C. The optimal dilution of the Cy3 secondary antibody was 600-fold with an incubation time of 45 min at 37 °C. The signal to noise ratios for each sample was determined by testing 20 sera negative for all the fusion proteins.

#### Cut-off value for determination of four viral antibodies

Twenty serums known to be negative for CSFV, PPV, JEV and PRRSV were 50-fold diluted and then were incubated with the prepared protein chips for 1 h at 37 °C. The CaptialBio Luxscan-10 K/A chip scanner was used to calculate the SNR value (median signal intensity to background median). Dates are shown in Table 1. The results show that when the SNR value of CSFV, PPV, JEV and PRRSV exceeded 1.78, 2.35, 3.38, and 1.28, respectively, the sample can be judged as antibody positive, otherwise antibody negative.

**Table 1:** Cut-off value for determination of four viral antibodies

	CSFV	PPV	JEV	PRRSV
SNR	1.78	2.35	3.38	1.58
Positive criteria	SNR > 1.78	SNR > 2.35	SNR > 3.38	SNR1.58
Negative criteria	SNR < 1.78	SNR < 2.35	SNR < 3.38	SNR < 1.58

#### Specificity, repeatability, and stability

**Fig. 4:**
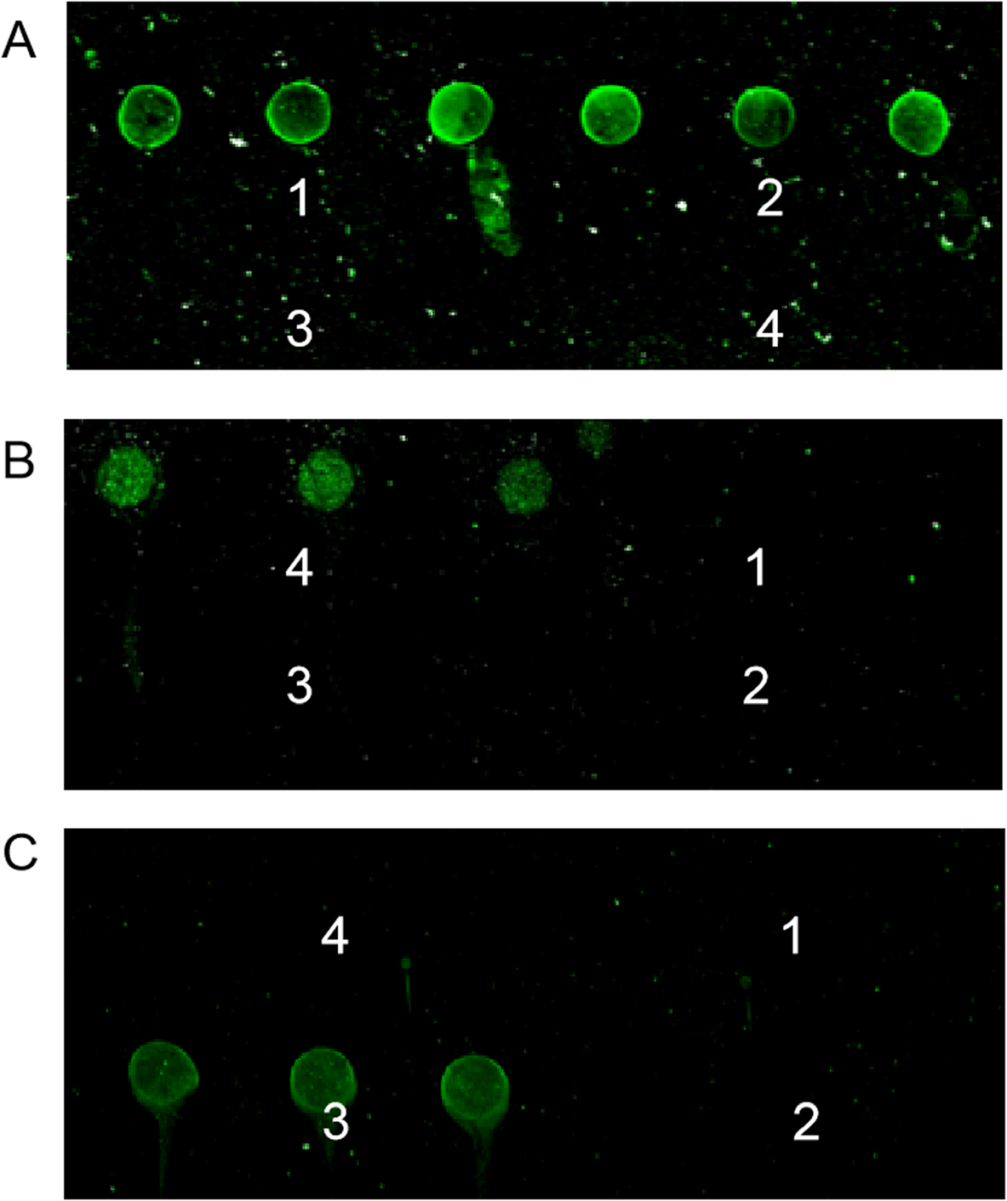
Specificity of the chip assay. Proteins were printed in triplicate in an array (1, CSFV-E2; 2, PRRSV-N; 3, JEV-EDIII; 4, PPV-VP2) and each array was printed in triplicate. **a** Incubated with CSFV & PRRSV positive serum; **b** Incubated with PPV positive serum; **c** Incubated with JEV positive serum

The four fusion proteins were printed at their optimized concentration in triplicate in an array, and the array was printed in triplicate. Each array was incubated with a specific protein-positive serum. The resulting fluorescent spots were protein specific as can be seen in Fig. 4. The results revealed that the arrays incubated with CSFV, PPV, JEV and PRRSV-positive sera showed fluorescent spots only at the corresponding protein positions.

Fifty clinical serum samples were tested using the chips under the optimized conditions; the test was done in triplicate with three technical repeats per test. The results showed that the coefficients of variation within a test were less than 2.5%, and the coefficients of variation between tests were less than 3% (Table 2), demonstrating the repeatability and stability that the chip assay.

**Table 2:** Stability of the protein chip

	Intra-batch repeat					Repeat between batches				
	1st group	2nd group	3rd group	Standard deviation	Coefficient of variation(%)	1st group	2nd group	3rd group	Standard deviation	Coefficient of variation(%)
CSFV	1.83	1.86	1.9	0.035	1.88	1.89	1.93	1.83	0.05	2.67
PPV	3.13	3.2	3.09	0.056	1.74	3.15	3.25	3.22	0.05	1.6
JEV	3.54	3.69	3.65	0.077	2.14	3.66	3.57	3.69	0.06	1.71
PRRSV	2.37	2.29	2.34	0.04	1.73	2.38	2.28	2.31	0.05	2.2

#### Comparison by method of seroprevalence in clinical samples

Two- hundred clinical samples were tested using the protein chip and commercial ELISA kits. The results demonstrate that for positive samples, the percent agreement between the methods was 100% for CSFV, 97.7% for PPV, 95.8% for JEV, and 100% PRRSV. For negative samples the agreement was 100, 86.2, 94.1, and 100%, respectively (Table 3).

**Table 3:** Comparison between the results of protein chip and commercial ELISA kit

	CSFV		PPV		JEV		PRRSV	
	Chip	ELISA	Chip	ELISA	Chip	ELISA	Chip	ELISA
Total (no.)	200		200		200		200	
Positive (no.)	183	183	175	171	120	115	193	193
Negative (no.)	17	17	25	29	80	85	7	7
Positive (%)	91.50%	91.50%	87.50%	85.50%	60.00%	57.50%	96.50%	96.50%
Negative (%)	9.50%	9.50%	12.50%	14.50%	40.00%	42.50%	3.50%	3.50%

## Discussion

With the increasing scale and density of the pig industry, the damage from pig infectious diseases, especially viral infectious diseases, is increasing. Classical swine fever [18], porcine parvovirus disease [19, 20], Japanese encephalitis [21] and porcine reproductive and respiratory syndrome [22] are serious viral diseases on large-scale pig farms in China. Moreover, pigs infected with these viruses are often infected with other pathogens as well. The mixed infection of diseases not only brings huge economic losses to the breeding industry but also reduces the immune effect of the herd [3]. Although diagnostic methods have been established for each of these viral infections, screening cannot be done simultaneously for these and other pathogens [6, 23, 24]. For diagnostics, protein chips are not yet widely used for animal diseases because of theirs high preparation costs [25, 26]. Our study expands the application of protein chips for diagnosing disease in pigs.

Early protein chips were of nitrocellulose or NC membrane (Nylon cellulose membrane) [17], due to the soft texture and space constraints of these materials, cross contamination often occurred between protein samples. Chip technology has continuously improved, and in this study, we used an epoxy chip. Epoxides react with various groups on the surface of proteins, such as hydroxyl, mercapto, carboxyl, without changing the properties of those proteins [27]. Compared with other chemically modified substrates, epoxy substrates have lower cost and are suitable for large-scale industrial production. In addition, compared with the ELISA assay, the biochip assay requires very low levels of proteins [28–30]. We used prokaryotically expressed CSFV-E2, PPV-VP2, JEV-EDIII, and PRRSV-N proteins as the capture antigens to simultaneously screen serum samples for antibodies to these viruses. Cy3-labeled secondary antibody was used to visualize the results. Our chip assay had very good sensitivity and specificity, and performed well in accordance with commercial ELISA kits. We screened 200 clinical samples using both the protein chip and commercial ELISA kits. The coincidence rates of the positive samples were 95.8 to 100% and the coincidence rates of the negative samples were 86.2 to 100%. We feel therefore, that our protein chip can replace ELISA kits as a diagnostic tool for identifying CSFV, PPV, JEV, and PRRSV infections.

## Conclusion

In summary, we have prepared a protein biochip that can be used to screen for antibodies in clinical serum samples after mixed infection. The data show that this protein biochip is specific and sensitive. This work has contributed to the development of high-density, integrated diagnostic biochips.

## Methods

### Plasmid construction

Using the sequences in GenBank as template, specific primer pairs (Table 4) were designed to amplify CSVF-E2 (FJ598612.1), JEV-ED III (LC095865.1), and PRRSV-N (KM252867.1). The three virus cDNAs stored in the laboratory were used to amplify the corresponding target genes. The amplicons were purified by AGE, digested by the restriction enzymes indicated in Table 1 and cloned into the corresponding sites of a pET-32a vector. The PPV VP2 gene (JQ710896.1) sequence (amino acids 156–438) was optimized using DNAStar software according to *E. coli* preference codon and then synthesized by the Jinkairui Company (Wuhan, China) (Table S1). The synthesized gene was cloned into a *Bam*HI*/Hind* III digested pET-32a vector. All constructs were verified by restriction digestion, PCR, and DNA sequencing.

**Table 4:** Primers used in the study

	Specific primer sequence(5′ → 3′)	Restriction sites	Amplification site	Fragment size
**CSFV**	CSFV-E2-F:5′-TGACTCTAGATATTTGGCATCATTGCATAAGGGGG-3′	XbaI	193–530	338 bp
	CSFV-E2-R:5′-TGACGGTACCGATCTTCATTTTCCACTGTGGTGG-3’	KpnI		
**JEV**	JEV-EDIII-F:5′-TGACGATATCCACCTGAAATGCAGGCTAAAAATGG-3’	EcoRV	849–1304	455 bp
	JEV-EDIII-R:5′-TGACAAGCTTGAATACCCCTCCAATGGAGCC-3’	HindIII		
**PRRSV**	PRRSV-N-F:5′-TGACGGATCCATGCCAAATAACAACGGCAGAC-3’	BamHI	1–372	372 bp
	PRRSV-N-R:5′-TGACCTCGAGTCATGCTGAGGGTGGTGTT-3’	XhoI		
**PPV**	PPV-VP2-F:5′-TGACGGATCCAGCGCAACCAGTCCG-3’	BamHI	amino acids 156–438	849 bp
	PPV-VP2-R:5′-TGACAAGCTTCATATTGCTTTTACCACCAATCGGA-3’	HindIII		

### Expression and purification of proteins

The expression and purification of these four fusion proteins was carried out as described previously [31–33]. Briefly, BL21(DE3) cells (TsingKe Biotechnology Co., Ltd., Beijing, China) were transformed with each recombinant plasmid, cultured 37 °C in LB (Luria Broth) medium supplemented with 100 μg/mL ampicillin until logarithmic phase (at OD_600_ of 0.6), then induced by IPTG at a final concentration of 0.2 mM (mmol/L) for 5 h at 37 °C. The re-suspended cells were lysed by sonication on ice (8 cycles × 1 min/cycle at intervals of 30 s), then centrifuged for 15 min at 10,000 x g. Supernatants and pellets (suspended in PBS (Phosphate buffered saline)) were collected, and analyzed by SDS-PAGE to access the solubility of each protein. Fusion proteins in inclusion bodies, were denatured overnight in 8 M urea at 4 °C, and remained in the supernatant after centrifugation. His-tagged fusion proteins were bound to HISTRAP HP (GE life , USA) and purified following the manufacturer’s instructions. The freshly purified proteins were treated with Detoxi-Gel™ Endotoxin Removing Gel (Thermo, USA) according to the manufacturer’s instructions, then aliquots and stored at − 80 °C. The protein concentration was measured by BCA protein quantification kit (Vazyme Biotechnology Co., Ltd., China).

### Preparation of the protein chip

**Fig. 5:**
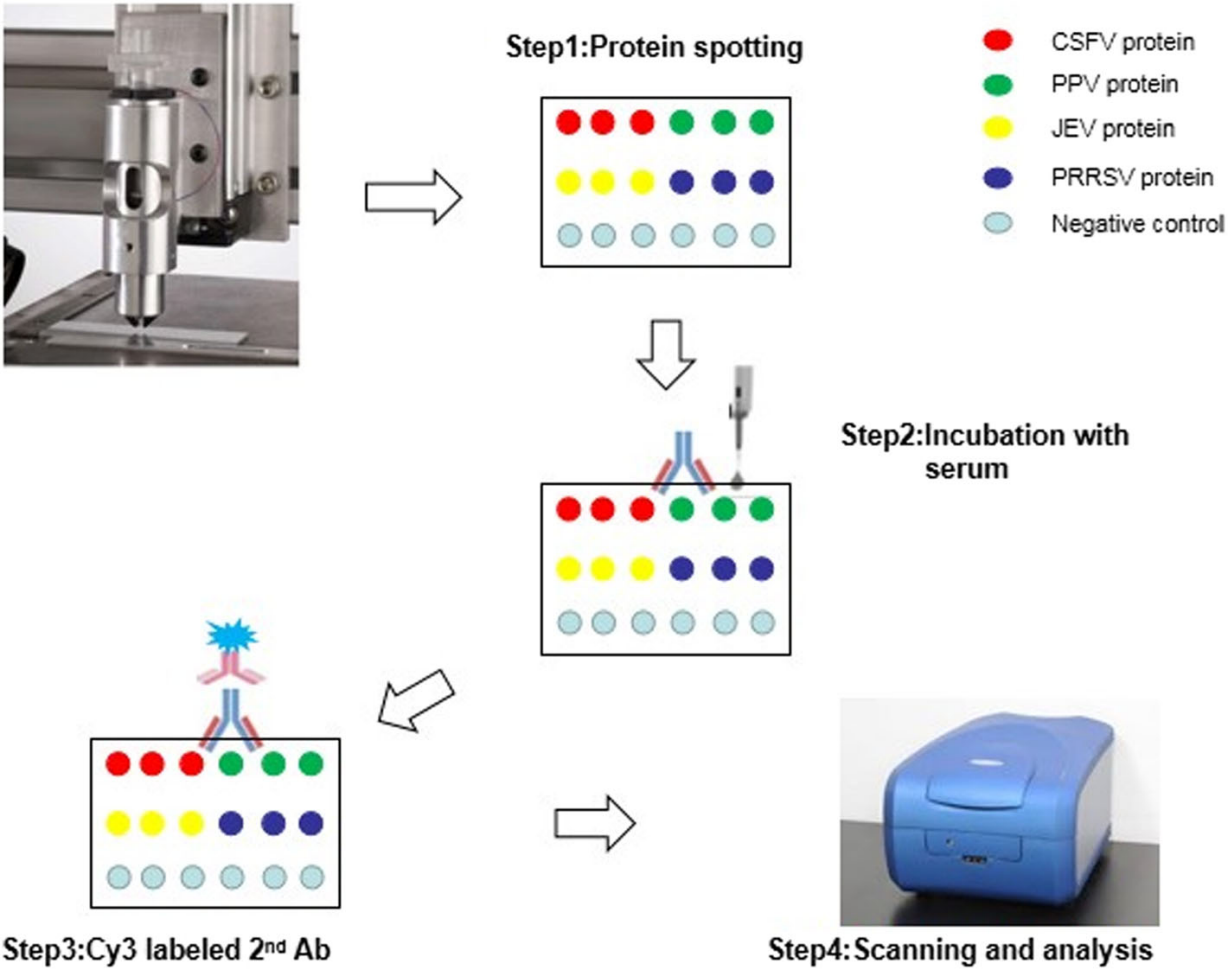
Schematic of chip assay. Step 1: According to the parameters of the chip matrix, protein samples in 80 μL of PBS/2% BSA were diluted to 0.4, 0.2, 0.1, 0.05, and 0.025 mg/mL. Each dilution of each protein was printed 3 times using the SmartArrayer™. Step 2: Chip was incubated with 150 μL of 1:50 fold dilution serum. Step 3: Chip was incubated with 100 μL of 1:600 Cy3 labeled goat anti-pig IgG, in darkness. Step 4: Scanning analysis was done with a Capital Bio Luxscan-10 K/A chip scanner

Figure 5 illustrates the following steps. Each purified protein in PBS/2% BSA was diluted to 0.4, 0.2, 0.05, and 0.025 mg/mL with 2X printing buffer (Capital Biotechnology Co., Ltd. Company, China). Each dilution of each protein was printed in triplicate onto the chip using a SmartArrayer™ microarray spotter (Capital Biotechnology Co., Ltd. Beijing, China). The chips were placed in an incubator overnight at 37 °C for 10 h. The chips were then washed with PBST (Phosphate buffered saline-Twen-20) and distilled water for 5 min each, then blocked for 2 h at 37 °C with 2% BSA (Shanghai Biotech Biotechnology Co., Ltd., China) in PBS. After washing with PBST, the chip was placed in a drying tube and centrifuged at 1000 rpm for 5 min, then incubated with clinical serum (Shengtaiyuan Agriculture and Animal Husbandry Development Co., Ltd. Nanjing,China) and incubated for 1 h at 37 °C. The chips were washed again and incubated with 1:600 Cy3 labeled goat anti-pig IgG (Immune Jackson, 138,102, USA) for 45 min at 37 °C in darkness. Chips were washed again three times in PBST then scanned and analyzed with a CapitalBio Luxscan-10 K/A chip scanner.

### Parameter optimization

#### Concentration of antigen

In serological assays, antigen concentration has a great impact on the specificity and sensitivity of the reaction; high signal to noise ratios are the result of suboptimal concentrations. Each of the purified proteins were diluted to 0.4, 0.2, 0.1, 0.05, and 0.025 mg/mL with 2 × printing buffer, 2% BSA was the negative control, then spotted onto the epoxy-based slides (Capital Biotechnology Co., Ltd. Beijing, China).

#### Concentration of printing buffer

The concentration of printing buffer affects whether a stable protein spot can be formed. Each of the purified proteins were diluted with PBS containing 40% or 50% vol commercial printing buffer and 50% homemade buffer (PBST + 20% glycerin).

#### Dilution of primary antibody and incubation time

Positive sera against CSFV, PPV, JEV or PRRSV were diluted 2, 5, 10, 50 and 100-fold in PBST and incubated with chips for 1 or 2 h at 37 °C to determine the best dilution of primary antibody and incubation time.

#### Dilution of secondary antibody and incubation time

Cy3 labeled fluorescent secondary antibodies were diluted 100, 200, 500, 600 and 800-fold, and incubated with chips for 30, 45, or 60 min at 37 °C to determine the optimal dilution and incubation time.

#### The cut-off value of detection

Twenty serums known to be negative for CSFV, PPV, JEV and PRRSV were 50-fold diluted and then were incubated with the prepared protein chips for 1 h at 37 °C. The CaptialBio Luxscan-10 K/A chip scanner was used to calculate the SNR value (median signal intensity to background median) and determine the cut-off value for detection of each viral protein.

### Specificity and repeatability

The four fusion proteins were printed in triplicate, at their optimized concentration, in an array and the array was printed in triplicate. Each array was incubated with a specific protein-positive serum. In addition to 50 clinical serum samples were tested three times, each test contained three technical repeats.

### Method caparison using clinical serum samples

Two-hundred pig serum samples were tested using the optimized conditions of the protein chip and commercial ELISA kits according to the manufacturer’s instructions. (CSFV and PRRSV ELISA kits; IDEXX, USA); PPV and JEV ELISA kits;(Keqian Biological Co., Ltd., Wuhan, China).

## Supplementary information

**Additional file 1 Table S1**. The optimized gene sequence of PPV-VP2

## Data Availability

Raw data is available from the corresponding author on reasonable request.

## Abbreviations

IPTG: Isopropyl β-D-1-Thiogalactopyranoside
ELISA: Enzyme-linked immunosorbent assay
PCR: Polymerase chain reaction
vol: Volume
BSA: Bovine serum albumin
NC: Nylon cellulose membrane
mM: mmol/L
PBS: Phosphate buffered saline
PBST: Phosphate buffered saline-Twen-20
LB: Luria Broth
